# Meningococcal Outer Membrane Protein NhhA Triggers Apoptosis in Macrophages

**DOI:** 10.1371/journal.pone.0029586

**Published:** 2012-01-04

**Authors:** Mikael Sjölinder, Georg Altenbacher, Matthias Hagner, Wei Sun, Sophia Schedin-Weiss, Hong Sjölinder

**Affiliations:** 1 Department of Genetics, Microbiology and Toxicology, Stockholm University, Stockholm, Sweden; 2 Department of Medical Biochemistry and Microbiology, Uppsala Biomedical Centre, Uppsala University, Uppsala, Sweden; Health Protection Agency, United Kingdom

## Abstract

Phagocytotic cells play a fundamental role in the defense against bacterial pathogens. One mechanism whereby bacteria evade phagocytosis is to produce factors that trigger apoptosis. Here we identify for the first time a meningococcal protein capable of inducing macrophage apoptosis. The conserved meningococcal outer membrane protein NhhA (*Neisseria* hia/hsf homologue A, also known as Hsf) mediates bacterial adhesion and interacts with extracellular matrix components heparan sulphate and laminin. Meningococci lacking NhhA fail to colonise nasal mucosa in a mouse model of meningococcal disease. We found that exposure of macrophages to NhhA resulted in a highly increased rate of apoptosis that proceeded through caspase activation. Exposure of macrophages to NhhA also led to iNOS induction and nitric oxide production. However, neither nitric oxide production nor TNF-α signaling was found to be a prerequisite for NhhA-induced apoptosis. Macrophages exposed to wildtype NhhA-expressing meningococci were also found to undergo apoptosis whereas NhhA-deficient meningococci had a markedly decreased capacity to induce macrophage apoptosis. These data provide new insights on the role of NhhA in meningococcal disease. NhhA-induced macrophage apoptosis could be a mechanism whereby meningococci evade immunoregulatory and phagocytotic actions of macrophages.

## Introduction

The pathogenic bacterium *Neisseria meningitidis* is a leading cause of bacterial meningitis and septicemia. During asymptomatic carriage meningococci colonise the nasopharynx but under certain conditions bacteria spread to the bloodstream and meninges. Disease progression is rapid and associated with high mortality. *Neisseria* hia/hsf homologue (NhhA) is an outer membrane protein homologous to the Hia and Hsf adhesins of *Haemophilus influenzae* [1]. A strain survey showed that the NhhA gene was present in all (85) strains tested and immuno-electron microscopy demonstrated the presence of NhhA on the outer membrane [1]. In a screen for vaccine candidates against serogroup B meningococci, NhhA was identified as a surface-exposed antigen that could be used to produce an antibactericidal antibody response in mice [2]–[3]. The C-terminal domain of NhhA is able to trimerize and form a translocator domain that allows localization of the protein to the bacterial surface [4]. In agreement, a C-terminal point mutation was shown to block trimerisation of NhhA [5]. *E. coli* expressing recombinant Nhha can bind to epithelial cells and furthermore direct interaction between purified NhhA and laminin as well as heparan sulphate was observed [4]. Meningococci lacking NhhA are more sensitive to complement-mediated bacterial killing in human serum and can be more easily phagocytosed by macrophages. In addition, NhhA promotes the interaction between capsulated, wildtype meningococci and macrophages [6]. Using a mouse model of meningococcal disease, Nhha was found to be of importance for colonization of nasal mucosa. Bacterial survival upon intranasal administration could only be observed when using wildtype meningococci and not with meningococci lacking NhhA [6].

Programmed cell death of host cells can be triggered by the release of proapoptotic factors from pathogenic bacteria or exposure of proapoptotic factors on the bacterial surface. Several species of bacteria can induce apoptosis such as *Pseudomonas aeruginosa* [7] and enteropathogenic *E. coli* [8]. Apoptosis can play an important role during bacterial infections and be either induced or inhibited by bacterial factors under different pathological conditions. In *N. meningitidis*, the ability of highly pathogenic strains and isolates to cause invasive disease and tissue damage was found to be closely linked to pro-apoptotic activity [9]. In contrast, strains associated with asymptomatic carriage did not induce apoptosis [9]. The bacterial membrane component lipopolysaccharide (LPS) and related compounds such as lipooligosaccharide (LOS) of *N. meningitidis* are important apoptosis-inducing factors [10]. However, meningococci lacking LOS can also provoke proinflammatory responses [11] and induce apoptosis [9]. The *N. meningitidis* porin PorB has been reported to prevent apoptosis in B cells, Jurkat T cells and HeLa epithelial cells by translocating to and interacting with mitochondria [12]. Interestingly, contradictory reports claim that the closely related PorB of *Neisseria gonorrhoeae* causes calcium influx and apoptosis in HeLa cells and U937 monocytes [13]–[14]. In addition, *norB* and *cycP*, two *N. meningitidis* genes involved in nitric oxide metabolism, were found to play a role in preventing nitric oxide-dependent apoptosis during infection [15].

Here we demonstrate that the meningococcal outer membrane protein Nhha not only plays an important role in promoting bacterial adhesion. Macrophages are of major importance for the host defense during meningococcal infections. We found that macrophages undergo apoptosis in the presence of NhhA.

## Results

### NhhA binds to macrophages

**Figure 1:**
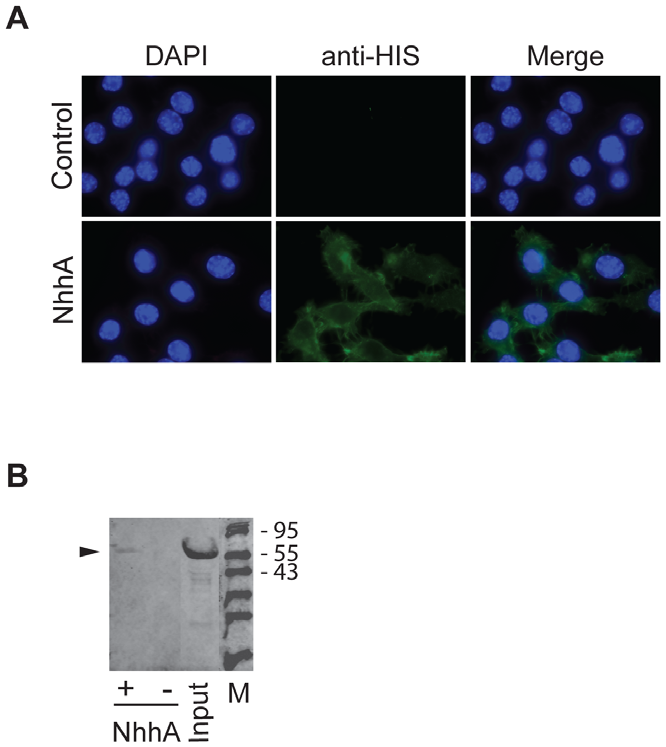
NhhA binds to macrophages. **A**: The RAW 264.7 mouse macrophage cell line was grown on coverslips and incubated with 400 nM recombinant NhhA for 4 hours. Immunofluorescence analysis was performed using a monoclonal mouse anti His-tag antibody followed by an Alexa Fluor 488-labeled anti-mouse antibody. Control cells were incubated with an unrelated His-tagged protein (NMC0101). One representative experiment of three is shown. **B**: Macrophages were incubated with 400 nM recombinant NhhA (+ NhhA) or NMC0101, an unrelated His-tagged protein (− NhhA) for 4 hours. Cells were washed in PBS, dissolved in SDS-PAGE sample buffer and cell-bound material or input material was analysed by immunoblot analysis using an anti His-tag antibody. Arrowhead indicates migration of NhhA. The sizes in kDa of a protein ladder is indicated. One representative experiment of three is shown.

In order to perform functional studies of NhhA we expressed NhhA in soluble form by excluding the C-terminal membrane anchoring domain. A sequence encoding amino acids 51 to 510 of NhhA from FAM20, a *N. meningitidis* serogroup C strain, was cloned into the vector pET-21a and expressed in *E. coli* in fusion with a histidine tag. The N-terminal of NhhA contains a predicted leader peptide that was excluded from the NhhA construct. NhhA was purified by affinity chromatography followed by gel chromatography to avoid endotoxin contamination. The recombinant NhhA was present as a monomer and did not undergo any detectable trimerisation (results not shown). Next, the ability of NhhA to bind to macrophages was investigated. RAW 264.7, a mouse macrophage cell line, was cultured under standard conditions and NhhA or an unrelated His-tagged control protein was added to the cell media. By immunofluorescence staining and immunoblotting we could detect binding of NhhA but not the control protein to macrophages (Fig. 1).

### NhhA induces apoptosis in macrophages

**Figure 2:**
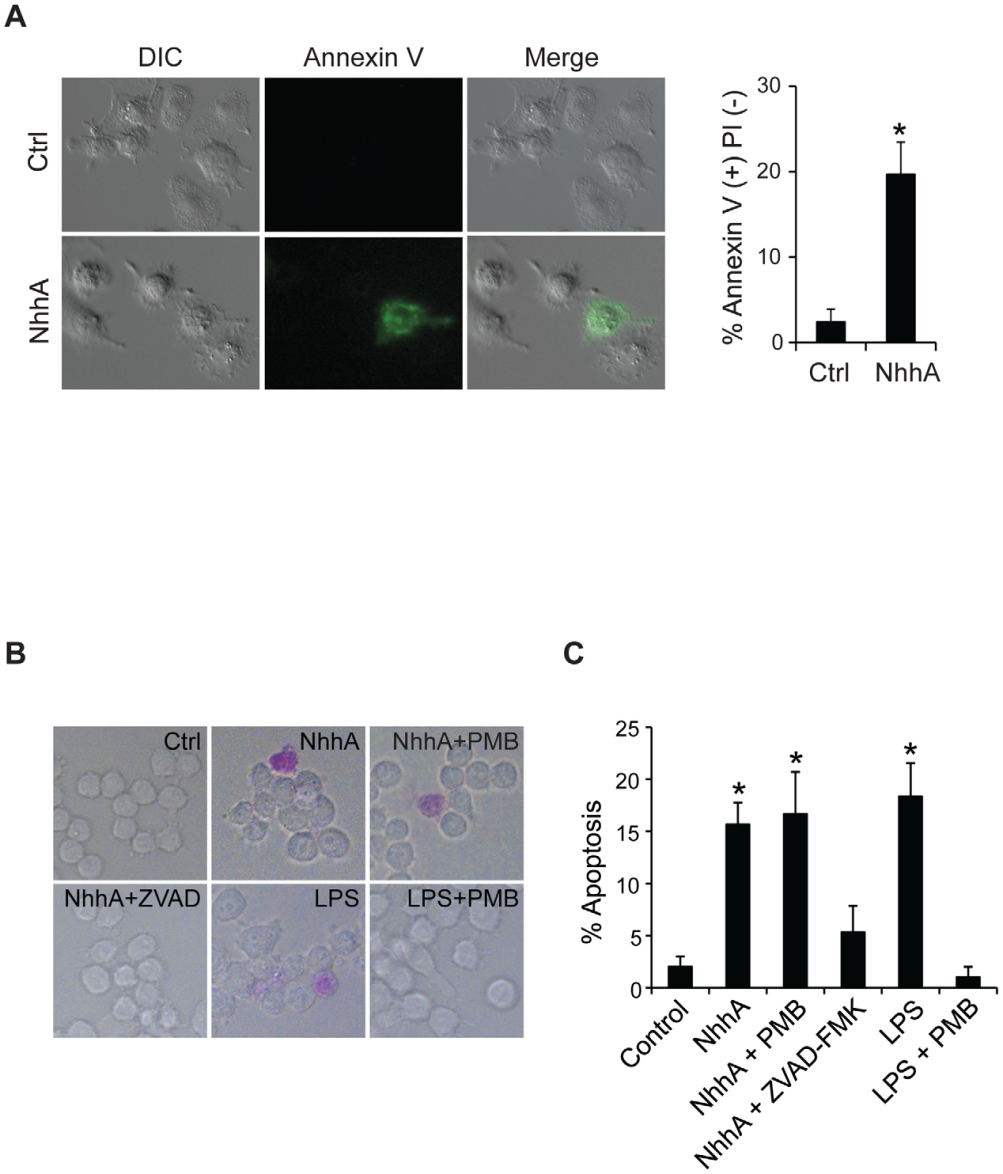
NhhA induces caspase-dependent apoptosis in macrophages. **A**: The RAW 264.7 mouse macrophage cell line was incubated with or without 400 nM recombinant NhhA and apoptotic cells were visualized by staining with an AnnexinV antibody and PI. The relative number of AnnexinV-positive and PI-negative cells were determined by fluorescence microscopy. **B**: Cells were incubated with NhhA as above or with 0.5 µg/ml *E. coli* LPS for 20 hours. If indicated, cells were pretreated with 1 µM Polymyxin B (PMB) or with the caspase inhibitor ZVAD-FMK (1 µM) for 1 h. Apoptotic cell were visualized using the APOPercentage kit. The relative numbers of apoptotic cells were determined using light microscopy by counting at least 100 cells per sample. Values indicate mean±SD of three independent experiments. *, p<0.05 versus untreated control.

**Figure 3:**
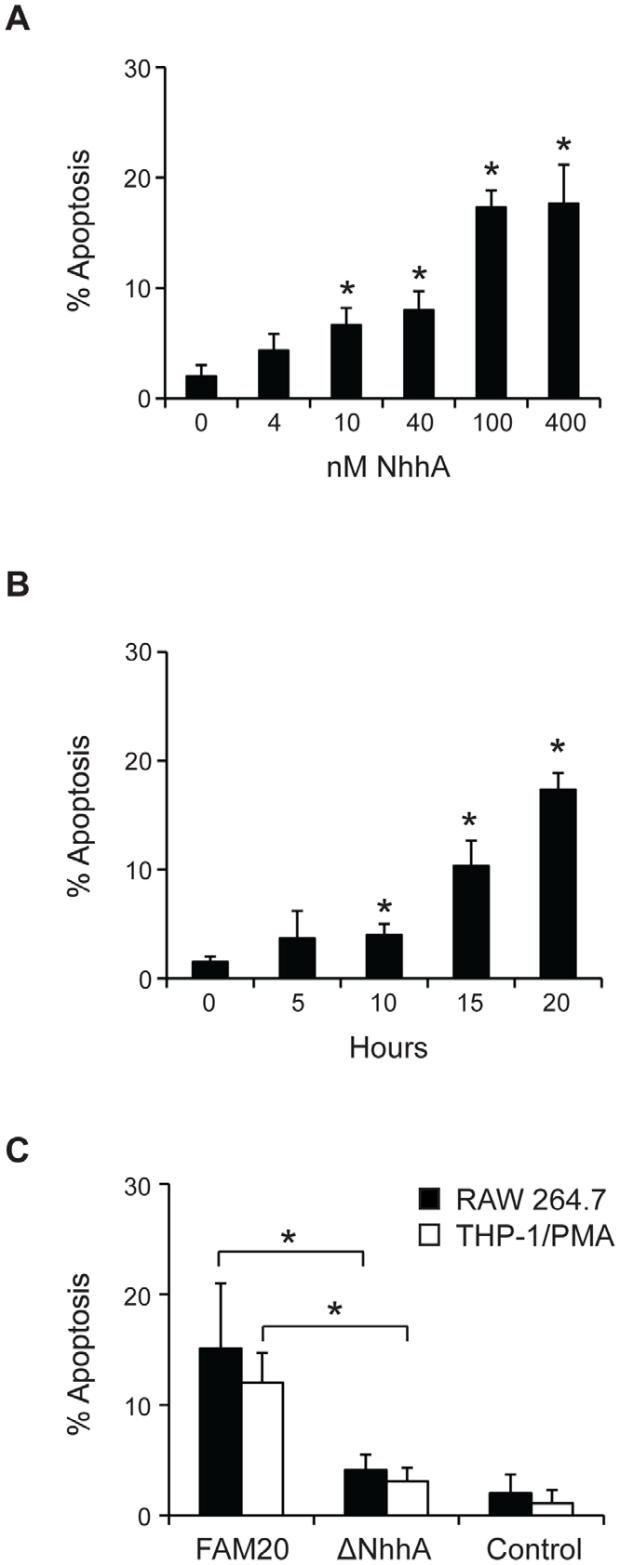
Effect of recombinant and endogenous NhhA on macrophage apoptosis. **A**: The RAW 264.7 mouse macrophage cell line was incubated with 0–400 nM recombinant NhhA for 20 hours. **B**: RAW 264.7 cells were incubated with 400 nM NhhA for 0–20 hours. **C**: RAW 264.7 or PMA-differentiated THP-1 cells were infected with the *N. meningitidis* serogroup C strain FAM20 or a NhhA-deficient mutant of FAM20 (ΔNhhA) at MOI = 100 for 20 hours. Control cells were uninfected. Apoptotic cells were visualized using the APOPercentage kit and the relative numbers of apoptotic cells were determined by light microscopy. Values indicate mean±SD of three independent experiments. *, p<0.05 versus untreated control(panel A+B) *, p<0.05 versus FAM20 treated cells (panel C).

We were interested to investigate if NhhA might have additional functions besides mediating bacterial adhesion to host cells. In initial experiments we noticed that exposure of macrophages to NhhA induced morphological changes associated with apoptosis such as blebbing and condensed and fragmented nuclei. To confirm an increased rate of apoptosis we stained NhhA-exposed cells with an AnnexinV antibody in combination with PI (Fig. 2A). Around 20% of the cells exposed to NhhA were found to stain positively for AnnexinV but not PI (Fig. 2A). Less than 2% of the NhhA-treated cells stained positively for PI but not AnnexinV (results not shown) indicating that NhhA-induced cell death proceeded mainly through apoptosis and not necrosis. To further study the capacity of NhhA to induce apoptosis we used a stain specifically labeling apoptotic cells exposing phosphatidyl serine on the cell surface (Fig. 2B). We found that after 20 h exposure to 400 nM NhhA, around 15% of the cells stained positively. The LPS antagonist Polymyxin B did not inhibit NhhA-induced apoptosis indicating that our preparations of purified recombinant NhhA were not contaminated with LPS. As expected, Polymyxin B inhibited LPS-induced apoptosis. In addition, the general caspase inhibitor ZVAD-FMK was found to inhibit NhhA-induced apoptosis indicating that NhhA triggers caspase-dependent apoptosis. The dose and time dependence of NhhA-induced apoptosis was investigated (Fig. 3). Already at a concentration of 10 nM NhhA the rate of apoptosis was significantly increased compared to an untreated control. An incubation time of 10 hours was required to induce statistically significant changes in the rate of apoptosis when using 400 nM NhhA. The reason that only a minority of the cells were found to be apoptotic after NhhA exposure could be that macrophages are generally considered to be relatively resistant to apoptosis. In addition, only cells with ongoing apoptosis that are still viable are detected. To further confirm the specificity of NhhA-induced apoptosis we cloned, expressed and purified an unrelated His-tagged meningococcal protein (NMC0101). NMC0101 failed to induce apoptosis in RAW 264.7 cells (results not shown).

In order to determine if endogenous meningococcal NhhA can play a role in regulating apoptosis we infected cells with the wildtype meningococcal strain FAM20 and with a FAM20 deletion mutant lacking NhhA [6]. We found that NhhA-deficient meningococci less efficiently induced apoptosis in macrophages, confirming that endogenous NhhA controls apoptosis in macrophages (Fig. 3C).

### NhhA specifically targets the monocyte/macrophage lineage

**Figure 4:**
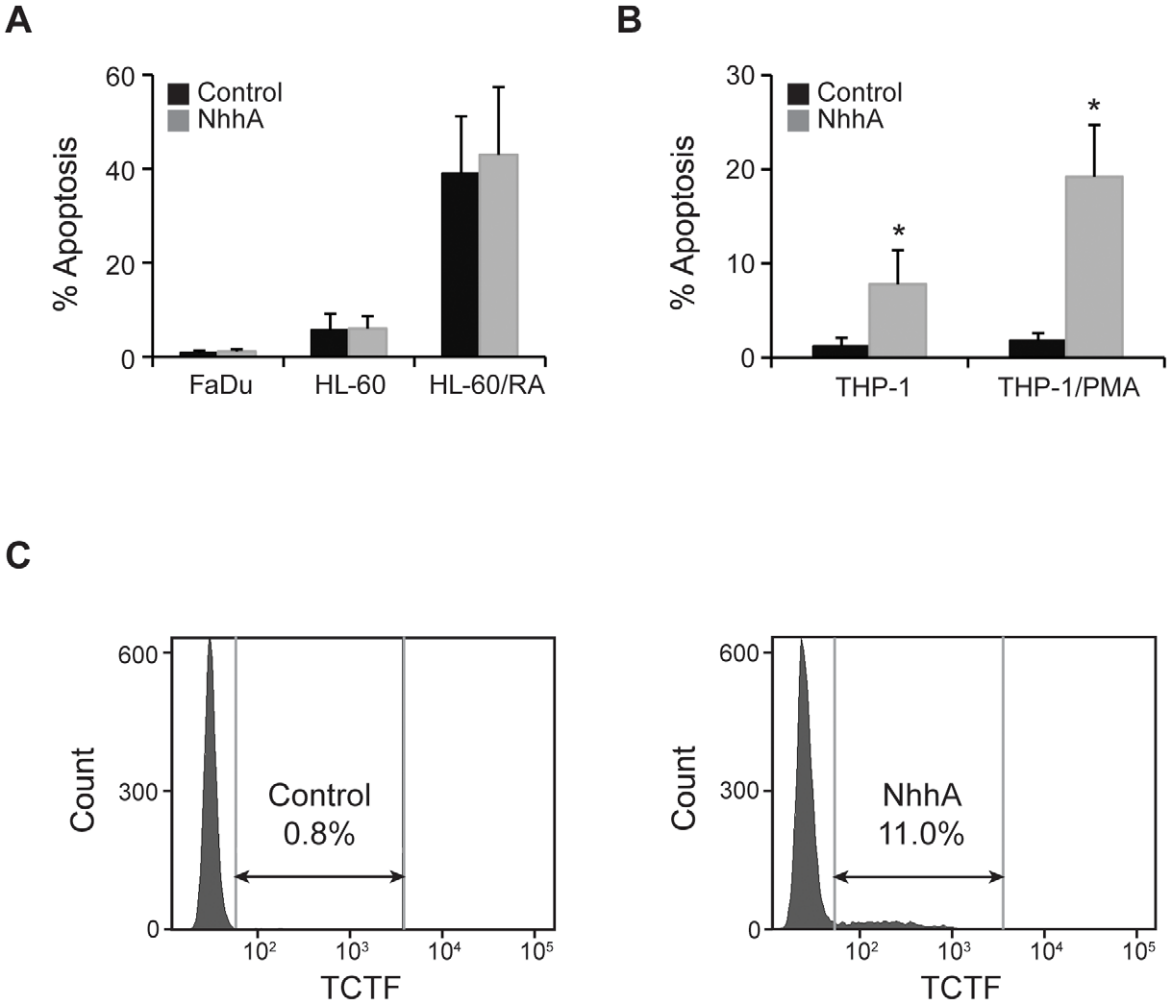
NhhA-induced apoptosis in different cell types. **A**: The pharyngeal epithelial cell line (FaDu) and the HL-60 promyelocytic leukemia cell line were exposed to 400 nM NhhA for 20 h. HL-60 cells were undifferentiated or differentiated to neutrophil-like cells by retinoic acid treatment (1 µM for 7 days, RA). **B**: The human monocytic cell line THP-1 was exposed to 400 nM NhhA. If indicated, THP-1 cells were differentiated to macrophages by treatment with PMA. Apoptotic cells were visualized using the APOPercentage kit and the relative numbers of apoptotic cells were determined by light microscopy. Values indicate mean±SD of three independent experiments. **C**: The human monocytic cell line THP-1 was exposed to 400 nM NhhA and apoptosis was measured by TCTF staining and flow cytometry. One representative experiment is shown.

In order to investigate if NhhA specifically acted on macrophages we also tested a human-derived pharyngeal epithelial cell line (FaDu) and human HL-60 promyelocytic leukemia cells. The HL-60 cells used in these experiments were undifferentiated or differentiated to a neutrophil-like phenotype using retinoic acid (Fig. 4A). In agreement with previous publications, retinoic acid-differentiated HL-60 cells were found to have a high level of background apoptosis [16]. NhhA did not alter the rate of apoptosis in any of these cells indicating that NhhA might specifically act on macrophages.

In order to investigate if human macrophages might be a target of NhhA we differentiated the monocytic cell line THP-1 to macrophage-like cells with phorbol 12-myristate 13-acetate (PMA). Undifferentiated and PMA-differentiated THP-1 cells were treated with NhhA and the relative number of apoptotic cells was determined. A significant increase in the number of apoptotic cells could be observed in undifferentiated as well as PMA-treated THP-1 cells (Fig. 4B). Differentiation to macrophages resulted in a striking increase in the sensitivity to NhhA-induced apoptosis indicating that macrophages might be the main target for NhhA-induced apoptosis. As a complement to the microscopy-based apoptosis assays we also analysed apoptosis in undifferentiated THP-1 cells by flow cytometry (Fig. 4C).

In order to further investigate the possibility of LPS contamination, NhhA was heated at 95° for 10 min. before treatment of PMA-differentiated THP-1 cells. Heat-treatment reduced the apoptosis-inducing activity of NhhA (400 nM) by 96.5±6.8%. In contrast, heat-treatment of LPS only reduced the apoptosis-inducing activity by 2.3±5.1%. Since LPS is highly resistant to heat inactivation [17] these data indicate that NhhA-induced apoptosis did not result from LPS contamination.

### Nitric oxide and cytokine signaling during NhhA-induced apoptosis

**Figure 5:**
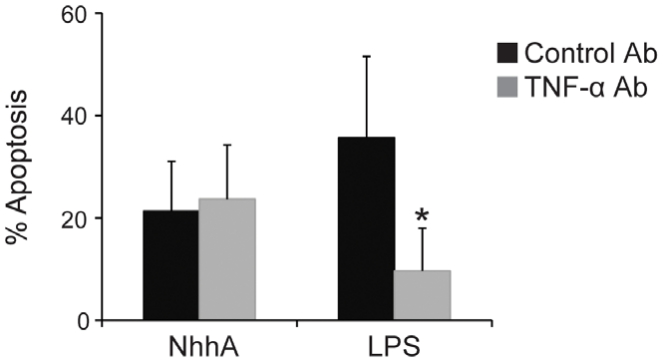
NhhA-induced apoptosis does not require TNF-α signaling. The RAW 264.7 mouse macrophage cell line was incubated with 400 nM recombinant NhhA or 0.5 µg/ml *E. coli* LPS for 20 hours. In addition, cells were incubated with an inhibitory TNF-α antibody or treated with an irrelevant isotype-matched antibody. Apoptotic cells were visualized using the APOPercentage kit and the relative numbers of apoptotic cells were determined by light microscopy. Values indicate mean±SD of three independent experiments. *, p<0.05.

Macrophages can produce the cytokine TNF-α that plays a role in proapoptotic signaling [10]. We investigated if NhhA-dependent release of TNF-α was of importance for induction of apoptosis by NhhA. However, the presence of a monoclonal antibody capable of blocking TNF-α bioactivity did not influence the proapoptotic effect of NhhA (Fig. 5). In contrast, and in agreement with previous studies [9], LPS-induced apoptosis was partially inhibited in the presence of the TNF-α antibody. We conclude that NhhA-induced apoptosis occurred independently of TNF-α signaling. We also investigated if TNF-α antibodies could block bacteria-induced apoptosis in PMA-differentiated THP-1 cells (Fig S1). We did not see any apoptosis-reducing effect of the antibodies suggesting that LOS might not play a leading role in meningococcal induction of macrophage apoptosis.

**Figure 6:**
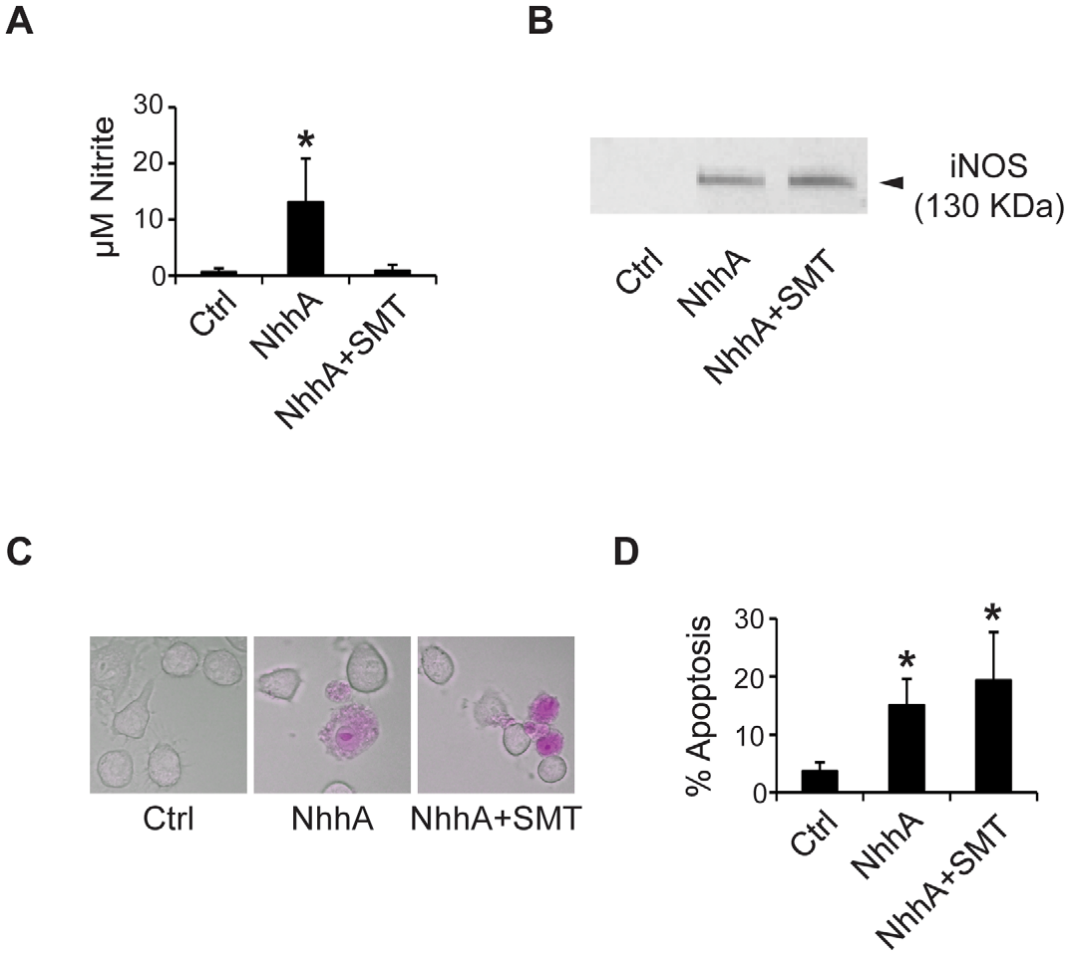
NhhA-induced apoptosis does not require NO. The RAW 264.7 mouse macrophage cell line was incubated with or without 400 nM recombinant NhhA for 20 hours. If indicated cells were pretreated for 1 h with the NO synthase inhibitor S-methylisothiourea (50 µM, SMT). **A**: Nitrite levels in cell supernatants were determined using Griess assay. **B**: The expression of iNOS was detected by immunoblot analysis. **C+D**: Apoptotic cells were visualized using the APOPercentage kit and the relative numbers of apoptotic cells were determined by light microscopy. Values indicate mean±SD of three independent experiments. *, p<0.05 versus untreated control.

Nitrosative stress has been reported to be involved in some forms of bacterially induced apoptosis [18]. We investigated if exposure of macrophages to NhhA induced nitric oxide (NO) synthesis. Nitrite, a stable NO metabolite, was measured using Griess assay. We found significantly increased levels of nitrite in NhhA-exposed cells as compared to untreated control cells (Fig. 6A). NhhA-induced nitrite production was inhibited by the Nitric Oxide Synthase inhibitor S-methylisothiourea (SMT). Production of NO was accompanied by induction of iNOS as judged by immunoblot analysis (Fig. 6B). Since NO production can contribute to proapoptotic signaling through the mitochondrial pathway we investigated if NO production was of importance for NhhA-induced apoptosis. However, we could not detect any change in the number of apoptotic cells when macrophages were pretreated with SMT before exposure to NhhA (Fig. 6D). Thus, NO production appears not to be of importance for NhhA-induced apoptosis in macrophages.

Since apoptosis-related release of the cytokine high mobility group box 1 (HMGB1) by macrophages is a crucial mediator of organ damage in sepsis [19] we determined if HMGB1 release is altered during apoptosis induced by wildtype and NhhA-deficient meningococci (Fig S2). We observed statistically significant decreases in the extracellular levels of HMGB1 when cells were exposed to NhhA-deficient bacteria as compared to wildtype bacteria.

## Discussion

The results presented here provide new insights on the role of NhhA during host-pathogen interaction. We found that NhhA induced caspase-dependent apoptosis in mouse and human macrophages, and to a lesser extent in monocytes. In contrast, endothelial cells and other myeloid cells appeared not to be targets of NhhA. Phosphatidylserine translocation to the outer layer of the cell membrane, a hallmark of apoptosis, was detected by two independent methods. A highly increased rate of apoptosis could be observed and after 20 hours exposure to 400 nM NhhA around 20% of the cells were found to be apoptotic. A significant increase in the number of apoptotic cells was observed already at 10 nM NhhA. In order to confirm that endogenous meningococcal NhhA regulates apoptosis we infected cells with wildtype and NhhA-deficient meningococci. We found that NhhA-deficient meningococci less efficiently induced apoptosis in macrophages, confirming that endogenous NhhA controls apoptosis in macrophages.

It was recently shown that NhhA can bind to vitronectin and thereby increase serum resistance by regulating complement activity [20]. Since vitronectin is abundant in serum the vitronectin-NhhA interaction could potentially regulate other actions of NhhA, such as its macrophage apoptosis-inducing activity described here. Bovine vitronectin binds to NhhA with similar affinity as human vitronectin [20]. Since our apoptosis experiments were carried out in the presence of bovine serum, we draw the conclusion that NhhA-induced macrophage apoptosis is not prevented by vitronectin. Further studies will be needed to elucidate to what extent the different functions of NhhA influence each other.

Macrophages produce TNF-α, a cytokine involved in proapoptotic signaling [10]. Meningococcal LOS induces TNF-α release which in turn contributes to induction of apoptosis in host cells [9]. A novel mechanism whereby meningococci regulate apoptosis was recently described, it was shown that strains associated with asymptomatic carriage promote shedding of TNF-α receptors whereby TNF-α is chelated and proapoptotic signaling prevented [9]. However, inhibition of TNF-α bioactivity did not affect NhhA-induced apoptosis. It appears that in contrast to LOS-induced apoptosis, NhhA-induced apoptosis does not require autocrine or paracrine TNF-α signaling. Instead, NhhA-induced apoptosis might involve other caspase-dependent pathways such as the intrinsic/mitochondrial pathway or the FasL or TRAIL pathways [21]. Exposure of macrophages to NhhA also triggered induction of iNOS and production of NO. Pretreatment of cells with a NO synthase inhibitor blocked NO production but not NhhA-induced apopotosis. Therefore, NO production appears not to be required for NhhA-dependent apoptosis. NO production is of major importance for host-defense mechanisms against *N. meningitidis* [22] and other bacteria. Thus, NhhA-induced NO production might instead contribute to the bactericidal activity of macrophages and other cells.

Systemic meningococcal infections are associated with a strong proinflammatory response that by itself can contribute to tissue injury and mortality. Several publications have indicated a relationship between organ failure in sepsis and apoptosis [23]–[24]. In severe sepsis, high levels of the cytokine high mobility group box 1 (HMGB1) are accumulated leading to lethal organ failure due to epithelial cell dysfunction [25], [26], [27]. Macrophages appear to play a key role in these events by their ability to respond to the presence of apoptotic cells by releasing HMGB1 [19]. It was shown that HMGB1 is a critical cytokine mediator of organ damage in severe sepsis since monoclonal antibodies against HMGB1 protected against organ damage but did not prevent the accumulation of apoptotic cells in the spleen [19]. NhhA-mediated macrophage apoptosis might contribute to HMGB1 release and tissue damage during meningococcal sepsis.

In summary, we demonstrate that NhhA promotes caspase-dependent macrophage apoptosis. NhhA-induced macrophage apoptosis might contribute to disease severity in meningococcal infections by reducing phagocytotic and immunoregulatory activities of macrophages.

## Materials and Methods

### Bacterial strains and growth conditions

FAM20, a *N. meningitidis* serogroup C strain, and its NhhA-deficient mutant [6], were grown at 37°C and 5% CO_2_ on GC medium base (Acumedia).

### Cloning and purification of recombinant NhhA

A sequence encoding amino acids 51 to 510 of NhhA was amplified by PCR using genomic DNA from FAM20, a *N. meningitidis* serogroup C strain, using the following primers; 5′-GCGAATGGATCCGATACCGATGAAGATG-3′ and 5′-CCTTTAAGCTTTGCGACGTTTGTAACATC-3′. After purification and digestion the PCR product was cloned into the vector pET-21a (Novagen) using restriction enzymes *Bam*HI and *Hind*III. Recombinant NhhA was expressed in *E. coli* in fusion with a C-terminal hexahistidine tag. NhhA was purified by affinity chromatography using Talon Resin (Clontech). Recombinant NhhA was then further purified by FPLC gel filtration. Purification was monitored by SDS-PAGE with Coomassie brilliant blue staining. The meningococcal protein NMC0101 was expressed and purified in the same way to serve as a control.

### Cell culture and treatment

Cell lines were obtained from American Type Culture Collection. The murine macrophage cell line RAW 264.7 and the human-derived pharyngeal epithelial cell line FaDu were cultured in DMEM plus 10% heat-inactivated fetal calf serum at 37°C and 5% CO_2_. The human monocytic cell line THP-1 and the human HL-60 promyelocytic leukemia cells were cultured in RPMI-1640 plus 10% heat-inactivated fetal calf serum at 37°C and 5% CO_2_. Retinoic acid treatment of HL-60 cells was with 1 µM all trans retinoic acid for seven days. THP-1 cells were differentiated to macrophages using 100 nM PMA (Sigma) for 3 days. Cells were maintained in serum-containing growth medium during the treatments described below. After seeding cells (50,000/ml) into 24- or 96-well cell culture plates or 8-well μ-slides (Ibidi), cells were allowed to attach for 6 h. If indicated, cells were pretreated with 1 µM Polymyxin B (InvivoGen), 1 µM caspase inhibitor ZVAD-FMK (BD Biosciences), 50 µM S-methylisothiourea (Sigma-Aldrich), 2.5 µg/ml anti TNF-α monoclonal antibody, clone MP6-XT22 (BD Biosciences) or 2.5 µg/ml of an unrelated isotype matched monoclonal antibody. After 1 h pretreatment cells were exposed to 0–400 nM NhhA for 5–20 h or 500 ng/ml *E. coli* LPS (Sigma-Aldrich) for 20 h. Alternatively, cells were exposed to wildtype FAM20 meningococci or a NhhA-deficient FAM20 mutant strain for 20 h at MOI = 100.

### Binding assay by Immunofluorescence microscopy

13 mm glass cover slips were coated with poly-L-lysine (5 µg/ml in PBS). RAW 264.7 cells were grown onto these over night, reaching approximately 40% confluence. The cells were then treated with 400 nM NhhA or an unrelated His-tagged protein for 4 hours. Unbound protein was removed by washing in PBS. Cells were then fixed in 2% paraformaldehyde for 15 min in RT, washed in PBS and permeabilized with 0.3% TritonX-100 in PBS for 10 min in RT. After washing in PBS, the cover slips were incubated in blocking buffer (3% BSA, 0.2% Tween, 1×PBS) for 30 min in RT. Cells were then incubated with primary antibody monoclonal mouse α-His-tag IgG2a (GE healthcare), diluted 1∶200, for 1 hour in RT. Incubation with Alexa Fluor 488-labeled secondary antibody (Invitrogen) diluted 1∶200 was performed for 1 hour in RT. The cover slips were fastened onto object glasses using ProLong® Gold mounting medium with DAPI (4′,6′-diamidino-2-phenylidole) (Invitrogen). Images were taken with an Axiovision Cell Observer HS fluorescent microscope (Carl Zeiss).

### Binding assay by Immunoblot analysis

RAW 264.7 cells were treated with 400 nM NhhA for 4 hours. The cells were washed in PBS, dissolved in SDS sample buffer, boiled at 95°C for 5 minutes and proteins were separated by SDS-PAGE (12%). Separated proteins were transferred to polyvinylidene floride membranes using a semidry transfer system (Bio-Rad). Membranes were blocked in 5% skim milk solution at 4°C o/n and incubated with primary antibody monoclonal mouse α-His-tag (GE healthcare), diluted 1∶1000 in PBS, at RT for 1 hour. Secondary antibody was an IRDye® 680 Goat anti-Mouse (1∶15,000, LI-COR Biosciences). For detection the Odyssey® Infrared Imaging System (LI-COR Biosciences) was used.

### Measurement of apoptosis

The relative number of cells undergoing apoptosis was determined using the APOPercentage kit (Biocolor) with the dye 3, 4, 5, 6-tetrachloro 2′, 4′, 5′, 7′ -tetraiodofluorescein (TCTF) following manufacturer instructions. Flow cytometry of TCTF-stained cells was done according to Meyer et al [28] using a LSRFortessa flow cytometer (BD biosciences). Alternatively cells were stained with propidium iodide (5 µg/ml) and a FITC-conjugated Annexin V antibody (BD Biosciences) according to manufacturer instructions. Cells were examined with an Axiovision Cell Observer HS fluorescent microscope (Carl Zeiss) and the relative number of cells that were Annexin V-positive and/or propidium iodide positive were determined.

### Measurement of NO production, iNOS expression and HMGB1 release

Production of NO was determined by measurement of nitrite in the cell supernatant using a Griess assay (Promega). iNOS expression was detected as follows. After washing twice with ice-cold PBS, cells were treated with lysis buffer containing 50 mM Tris-HCl (pH 7.6), 0.3 M Sucrose, 2 mM EDTA, 1 mM PMSF, 2 mM DTT, 20% Glycerol, 0.5% IGEPAL. The concentration was measured and equal amounts (30 µg) of protein was separated by SDS-PAGE and transferred to PVDF membrane (Santa Cruz Biotechnology). The primary antibody was a rabbit anti-mouse iNOS (1∶500, MyBioSource) and as second antibody IRDye® 680 Donkey anti-Rabbit (1∶15,000, LI-COR Biosciences) was used and detected with the Odyssey® Infrared Imaging System (LI-COR Biosciences). The levels of HMGB1 in cell supernatants was determined using an indirect ELISA assay with a mouse anti-HMGB1 antibody (Santa Cruz, SC-74085).

### Statistical analysis

Student's *t*-test was used for statistical analysis. Differences between means were considered significant if *P*≤0.05.

## Supporting Information

Figure S1 **TNF-α signaling during bacterially induced macrophage apoptosis.** The RAW 264.7 mouse macrophage cell line was infected with the *N. meningitidis* serogroup C strain FAM20 at MOI = 100 for 20 hours. In addition, cells were incubated with an inhibitory TNF-α antibody or treated with an irrelevant isotype-matched antibody. Apoptotic cells were visualized using the APOPercentage kit and the relative numbers of apoptotic cells were determined by light microscopy. Values indicate mean±SD of three independent experiments.
(TIF)

Figure S2 **Release of the cytokine HMGB1 after bacterially induced macrophage apoptosis.** PMA-differentiated THP-1 cells were infected with the *N. meningitidis* serogroup C strain FAM20 or NhhA-deficient meningococci at MOI = 100 for 20 hours. Release of the cytokine HMGB1 was examined by indirect ELISA. Values indicate mean±SD of three independent experiments. *, p<0.05.
(TIF)
