# Supplementary figures and images for: Meningococcal Outer Membrane Protein NhhA Triggers Apoptosis in Macrophages
Source: PLoS One. 2012 Jan 4;7(1):e29586. doi: 10.1371/journal.pone.0029586 (PMC3251587; doi:10.1371/journal.pone.0029586)

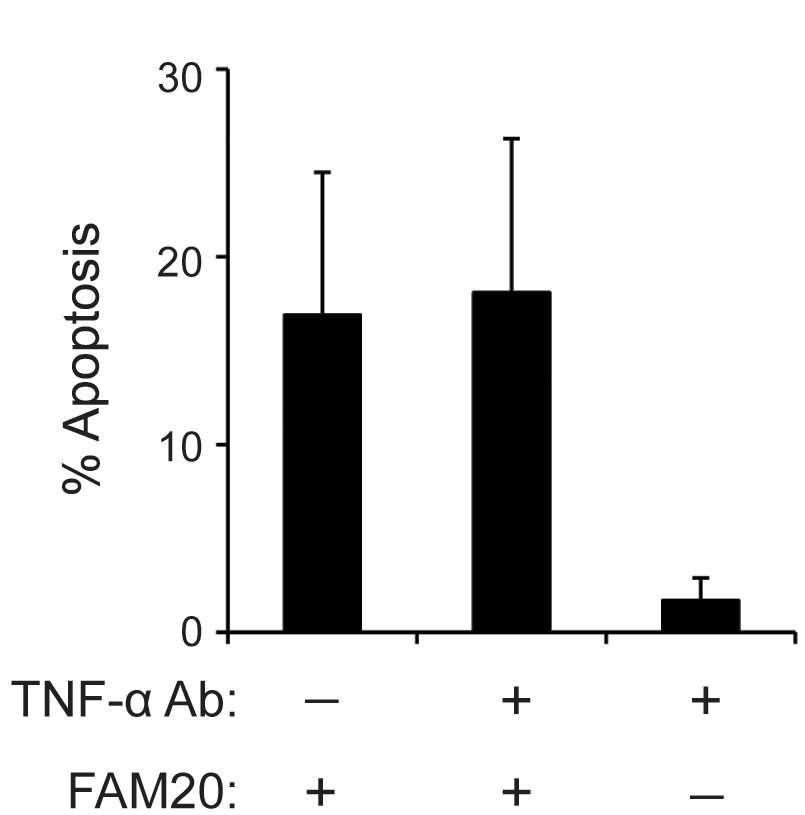

Supplement: Figure S1 — TNF-α signaling during bacterially induced macrophage apoptosis. The RAW 264.7 mouse macrophage cell line was infected with the N. meningitidis serogroup C strain FAM20 at MOI = 100 for 20 hours. In addition, cells were incubated with an inhibitory TNF-α antibody or treated with an irrelevant isotype-matched antibody. Apoptotic cells were visualized using the APOPercentage kit and the relative numbers of apoptotic cells were determined by light microscopy. Values indicate mean±SD of three independent experiments. (TIF) [file pone.0029586.s001.tif]

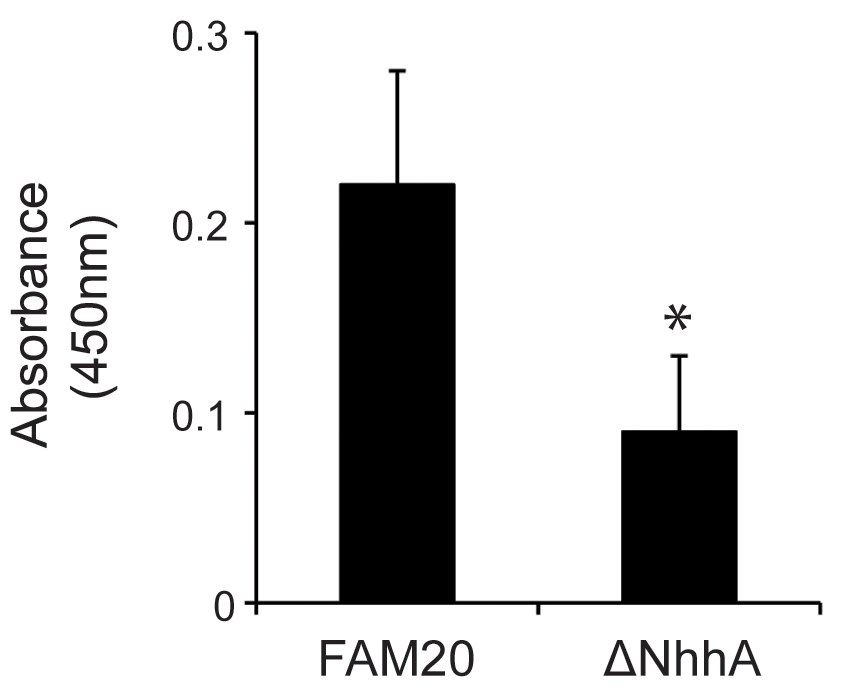

Supplement: Figure S2 — Release of the cytokine HMGB1 after bacterially induced macrophage apoptosis. PMA-differentiated THP-1 cells were infected with the N. meningitidis serogroup C strain FAM20 or NhhA-deficient meningococci at MOI = 100 for 20 hours. Release of the cytokine HMGB1 was examined by indirect ELISA. Values indicate mean±SD of three independent experiments. *, p<0.05. (TIF) [file pone.0029586.s002.tif]
